# Enhancing Radiation Shielding Efficiency of *Nigella sativa* Eumelanin Polymer Through Heavy Metals Doping

**DOI:** 10.3390/polym17050609

**Published:** 2025-02-25

**Authors:** Mohammad Marashdeh, Nawal Madkhali

**Affiliations:** Department of Physics, College of Sciences, Imam Mohammad Ibn Saud Islamic University (IMSIU), Riyadh 13318, Saudi Arabia; namadkhali@imamu.edu.sa

**Keywords:** eumelanin, mass attenuation coefficient, metal doping, mean free path, radiation protection efficiency

## Abstract

Gamma radiation shielding is necessary for many applications; nevertheless, lead creates environmental risks. Eumelanin, a natural polymer, is a viable alternative, although its effectiveness is limited to lower gamma-ray energy. This research looks at how doping the herbal eumelanin polymer (*Nigella sativa*) with heavy metals including iron (Fe), copper (Cu), and zinc (Zn) affects its gamma radiation shielding characteristics. The inclusion of these metals considerably increases the linear attenuation coefficient (*μ*) and mass attenuation coefficient (*μ_m_*) of eumelanin, especially at lower photon energies where the photoelectric effect is prominent. The *μ* value of pure eumelanin is 0.193 cm^−^^1^ at 59.5 keV. It goes up to 0.309 cm^−^^1^, 0.420 cm^−^^1^, and 0.393 cm^−^^1^ when Fe, Cu, and Zn are added, in that order. Similarly, the mass attenuation coefficients increase from 0.153 cm^2^/g for pure eumelanin to 0.230, 0.316, and 0.302 cm^2^/g for the Fe-, Cu-, and Zn-doped samples. At intermediate and higher energies (661.7 keV-to-1332.5 keV), where Compton scattering is the main interaction, differences in attenuation coefficients between samples are not as noticeable, which means that metal additions have less of an effect. The mean free path (MFP) and radiation protection efficiency (RPE) also show these behaviors. For example, at 59.5 keV the MFP drops from 5.172 cm for pure eumelanin to 3.244 cm for Mel-Fe, 2.385 cm for Mel-Cu, and 2.540 cm for Mel-Zn. RPE values also go up a lot at low energies. For example, at 59.5 keV Cu-doped eumelanin has the highest RPE of 34.251%, while pure eumelanin only has an RPE of 17.581%. However, at higher energies the RPE values for all samples converge, suggesting a more consistent performance. These findings suggest that doping eumelanin with Fe, Cu, and Zn is particularly effective for enhancing gamma-ray shielding at low energies, with copper (Cu) providing the most significant improvement overall, making these composites suitable for applications requiring enhanced radiation protection at lower gamma-ray energies.

## 1. Introduction

Ionizing radiation is an omnipresent aspect of modern life, emanating from both natural and artificial sources. While it has beneficial applications in medicine, industry, and research, it poses significant threats to human health and the environment. Exposure to ionizing radiation can lead to serious health problems, including cancer, damage to the nervous system, and mutations in human DNA [1,2]. According to the World Health Organization (WHO), even low doses of radiation can increase the risk of adverse health effects, making it crucial to develop effective protective measures against such exposures [3]. This underscores the need for robust radiation shielding materials, particularly in environments where ionizing radiation is prevalent, such as hospitals, research laboratories, and nuclear facilities.

Ionizing radiation finds applications across various sectors, including healthcare, agriculture, military, and industrial processes. In healthcare, for instance, radiotherapy uses ionizing radiation to treat cancer, targeting malignant cells while minimizing damage to surrounding healthy tissue [4,5,6,7,8,9]. In agriculture, radiation can be employed for pest control and food preservation, enhancing crop yields and extending shelf life [6]. Military applications include the use of radiation detection equipment for national security purposes, while industrial radiography is essential for the non-destructive testing of materials and components [7,8]. Despite these benefits, the potential risks associated with ionizing radiation necessitate effective shielding to protect both personnel and the public.

Radiation shielding materials serve a critical function in mitigating the harmful effects of ionizing radiation. These materials are employed in various forms, including structural components of buildings, protective suits for personnel, containers for radioactive materials, and windows in medical imaging equipment [9,10]. For example, radiation shielding is vital in diagnostic imaging procedures such as X-rays and computed tomography (CT) scans to protect patients and healthcare providers from unnecessary exposure [11]. Furthermore, in nuclear power plants effective shielding is necessary not only for the protection of workers but also to ensure the safety of surrounding communities [10].

Historically, metallic lead and lead-based compounds have been the primary materials used for shielding against X-rays and gamma rays due to their high density and atomic number, which make them effective at attenuating radiation [11]. However, the use of lead comes with significant drawbacks. Lead is a toxic and carcinogenic substance, and its corrosive properties can lead to environmental contamination [12]. Moreover, lead is ineffective against neutron radiation, producing high-energy secondary ionizing radiation when exposed. These limitations have prompted researchers to explore alternative materials that are non-toxic and environmentally friendly [13,14]. One promising candidate is eumelanin, a complex biological polymer that plays a crucial role in determining the color of skin, hair, and eyes in various organisms [15,16]. Eumelanin is produced through the melanogenesis process, which involves the polymerization of the amino acid tyrosine. Melanin is categorized into categories based on the molecular precursor from which the pigment is derived. Eumelanin (black melanin) consists primarily of oligomers of 5,6-dihydroxyindole (DHI) and 5,6-dihydroxyindole-2-carboxylic acid (DHICA) [17].

Beyond its aesthetic functions, eumelanin possesses unique physicochemical properties that have attracted considerable scientific attention in recent years. Notably, eumelanin is capable of absorbing a wide range of electromagnetic radiation, including ultraviolet (UV), visible, and infrared (IR) light [17,18]. This broad absorption spectrum not only supports its protective functions against UV radiation but also opens new avenues for its use in materials science, electronics, and biomedical engineering [19,20]. Natural eumelanin is a complex biopolymer that plays a crucial role in protecting organisms from harmful UV radiation and oxidative stress. Additionally, scientists explore the effects of metal modification on eumelanin’s optical properties after UV irradiation. This research shows that eumelanin, when doped with transition metals such as Fe, Co, and Zn, retains substantial absorption characteristics across a broad spectrum (200–500 nm). Density functional theory (DFT) analysis indicates that these metal modifications enhance eumelanin’s structural properties and optical reactivity, potentially contributing to treatments for skin diseases associated with excessive eumelanin secretion [18,21,22]. Recent studies have highlighted the potential of eumelanin as a bioinspired material for advanced technological applications. For instance, researchers have investigated its role in developing novel materials for solar energy harvesting and photoprotection [17]. Eumelanin’s ability to absorb and dissipate excess energy makes it an attractive candidate for various applications in nanotechnology and drug delivery systems [18]. Furthermore, its biocompatibility and biodegradability enhance its appeal in biomedical applications, where the safety and environmental impact of materials are of paramount importance [19]. The incorporation of metal elements into eumelanin has been shown to significantly enhance its physical and mechanical properties. Research indicates that metal-doped eumelanin exhibits improved mechanical strength, thermal stability, optical absorption, electrical conductivity, and radioprotective qualities [23]. For instance, the addition of metal ions such as silver, copper, and zinc has been shown to improve the radiation attenuation properties of eumelanin, making it a suitable candidate for use in radiation shielding applications. The enhanced characteristics of metal-doped eumelanin composites offer new opportunities for their integration into high-tech applications, including electronic devices, protective coatings, and specialized radiation shielding materials. The simulation results support the spatial arrangement hypothesis, which is that the organization of melanin in enclosure structure may give additional shielding. The arrangement of melanin on the cell membrane may limit the absorbed dose of highly energetic particles at the nucleus, but this impact is cumulative across the fungal colony. A lattice of melanin ghosts will provide consistent shielding for colony cells in an anisotropic radiation environment. The bulk Sepia melanin shielding effect towards the gamma radiation of 122–140 keV energies showed that 4 mm of melanin cut the dose by ~33% and the linear attenuation coefficient was calculated to be 1.01 cm^−1^ [24,25,26,27,28].

In the present study, we focus on synthesizing eumelanin composites using three metallic elements: iron (Fe), copper (Cu), and zinc (Zn). These metals were chosen due to their availability, cost-effectiveness, and potential for enhancing the radiation shielding properties of eumelanin. The synthesized eumelanin samples will undergo measurements of linear and mass attenuation coefficients within the photon energy range of 59.9 keV-to-1332 keV, which is critical for assessing their effectiveness against X-rays and gamma rays. We will compare our experimental results with those calculated using the XCOM program developed by Berger and Hubbell, which provides a reliable database for photon cross-sections over a wide energy range [29]. Additionally, this study will examine several key radiation shielding parameters, including the mean free path (MFP), radiation protection efficiency (RPE), half-value layer (HVL), and effective atomic number (*Z_eff_*) for the fabricated eumelanin samples. These parameters are essential for evaluating the suitability of the synthesized composites for radiation shielding applications. By systematically exploring these aspects, this research aims to contribute valuable insights into the potential of metal-doped eumelanin as a viable alternative to conventional radiation shielding materials.

## 2. Materials and Methods

### 2.1. Sample Preparation

Natural eumelanin was extracted from *Nigella sativa*, as mentioned in Ref. [18]. To this, 15 g of *Nigella sativa* eumelanin was treated with 100 mL of sodium hydroxide (2 M) and sonicated using an ultrasound device for 3 h at 100 °C. About 50 mL of hydrochloric acid (2 M) was distilled with a burette while stirring the concentrated melanin mixture and monitoring to adjust the pH to 6.5.

**Figure 1:**
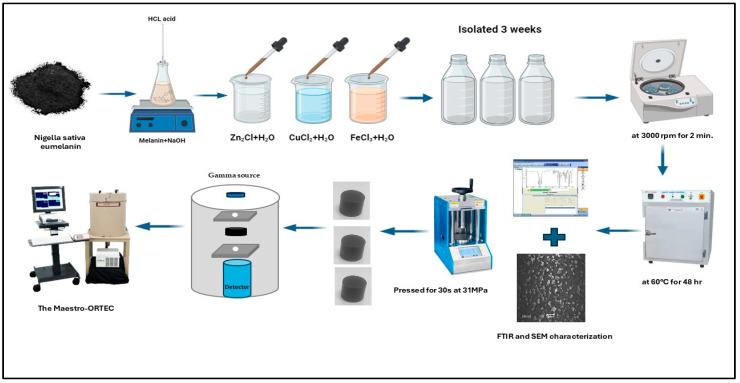
Schematic of the process followed for the synthesis of eumelanin doped with Zn, Fe, and Cu ions.

For the preparation of iron-doped eumelanin (Fe-Mel), iron(II) chloride (FeCl_3_) was added to the eumelanin solution in a 1:10 ratio. The resulting mixture was left in each sample for three weeks while the color changed from red to transparent in the melanin sample with iron chloride, red to transparent in the eumelanin sample with copper chloride, and white to transparent in the melanin sample with zinc oxide. Subsequently, the samples were centrifuged at 3000 rpm for 2 min, and then dried in an oven at 60 °C for 48 h. Similar procedures were followed to create copper-doped (Cu-Mel) and zinc-doped eumelanin (Zn-Mel) using CuCl_2_ and ZnCl_2_, respectively. All resulting compounds were all black in color. A PerkinElmer 580 B-IR spectrometer (industrialbid, Las Vegas, NV, USA) was used to obtain Fourier transform infrared (FTIR) spectra. The SERON-AIS 1800C scanning electron microscope (SEM), Seron Technologies Inc., Uiwang, Republic of Korea: was used to study the morphology and has a resolution of ∼20 µm. The APEX™ EDS software program was used for collecting and analyzing energy-dispersive X-ray spectroscopy (EDS/EDX). The synthesis process is illustrated in Figure 1. XRD measurements were conducted using a Discover diffractometer (Bruker (Billerica, MA, USA): D8 with Cu-Kα radiation, λ = 1.5406Å).

The samples were formed into discs after characterization by FTIR and SEM using a manual hydraulic press machine for 30 s at 31 MPa, as shown in Figure 1. The density of the samples was then determined by dividing their mass (in grams) by their volume (in cubic centimeters).

### 2.2. Evaluations of Gamma-Ray Shielding Parameters

Theory:

The primary parameter under investigation is the linear attenuation coefficient, *μ*, which can be calculated using Equation (1) [28].

(1) $$I=I_{o}{\;e}^{-\mu x}$$

where *x* represents the thickness of the sample measured in (cm) and *I* and *I_o_* denote the beam intensity after traversing the thickness and the initial intensity, respectively. The linear attenuation coefficient (*μ*) was used to calculate the mass attenuation coefficient (*μ_m_*) using the following formula:

(2) $$\mu_{m}=\frac{\mu }{\rho }$$

This formula ensures an accurate evaluation of attenuation properties among materials with varying densities by establishing an individual relationship between *μ* and $\mu_{m}$.

The error of the ∆ *μ_m_* measurement is calculated using Equation (3).

(3) $$\frac{\Delta \mu_{m}}{\mu_{m}}=\sqrt{{\left(\frac{\Delta \rho x}{\rho x}\right)}^{2}+{\left(\frac{\Delta I}{I}\right)}^{2}+{\left(\frac{\Delta I_{o}}{I_{o}}\right)}^{2}}$$

where ∆ ρx, ∆*I_o_*, and ∆*I* are the error of the mass density, unattenuated photon intensity, and attenuated photon intensity, respectively.

The half-value layer (HVL) is the thickness required to cut the amount of radiation in half and is calculated as shown in Equation (4) [30]:

(4) HVL=ln2μ

The mean free path (MFP), defined as the average distance between two consecutive gamma photon interactions, is measured in centimeters (cm) [31]. The mean free path can be determined using Equation (5):

(5) MFP=1μ

where *μ* is the linear attenuation coefficient in inverse centimeters (cm^−1^).

In addition, radiation protection efficiency (RPE) can be employed to evaluate the shielding effectiveness of composite samples based on the linear attenuation coefficient *μ*, as described by the following relation [32].

(6) $$\mathrm{RPE}=\left(1-e^{-\mu }\right)\times 100\;\;\;\;\;\left(\%\right)$$

The mass attenuation coefficients are used to calculate the total atomic cross-section, which can be determined using Equation (7).

(7) $$\sigma_{a}=\frac{{\left(\mu_{m}\right)}_{sample}}{N_{A}\sum_{i}^{n}\left(W_{i}/A_{i}\right)}$$

In this equation, *N_A_* represents Avogadro’s number, and *A_i_* represents the atomic Weight of the constituent element in the sample. The total electronic cross-section for the element can be found using Equation (8) [33].

(8) $$\sigma_{l}=\frac{1}{N_{A}}\overset{n}{\underset{i}{\sum }}\frac{f_{i\;\;}A_{i\;}}{Z_{i\;}}{\left(\mu_{m}\right)}_{i}$$

In this equation, fi represents the number of atoms of element I relative to the total number of atoms in the composite material, and *Z_i_* represents the atomic number of the ith element in the sample. The selection of a suitable material for radiation dosimetry and detection relies heavily on the effective atomic number (*Z_eff_*) and effective electron number (*N_eff_*). To obtain the compound’s effective atomic number (*Z_eff_*), one may calculate the ratio between the total atomic cross-section and the total electronic cross-section using Equation (9) [23].

(9) $$Z_{eff}=\frac{\sigma_{a}}{\sigma_{l}}$$

### 2.3. Experimental Setup

**Figure 2:**
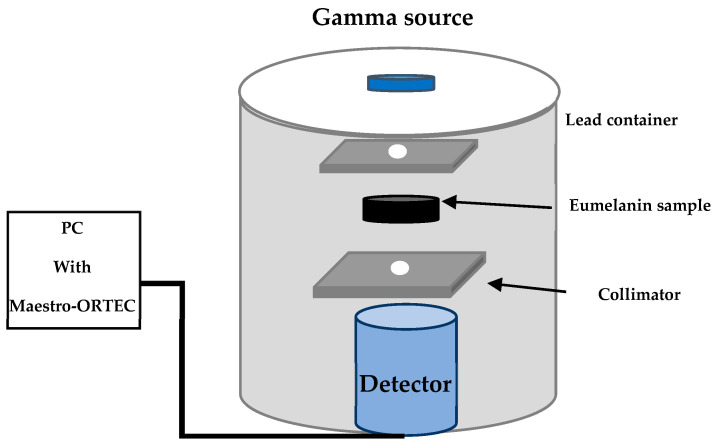
Schematic of the experimental setup.

The fabricated eumelanin samples were irradiated using three typical point sources: ^214^Am (59.5 keV), ^137^Cs (661.7 keV), and ^60^Co (1173.2 and 1332.5 keV). A sodium iodide (NaI (TI)) scintillation detector measuring 2” × 2” was employed to quantify the energy intensity. To protect the system from background radiation and scattering, a lead container was used. The Maestro-ORTEC (version 7.2) was utilized to analyze and identify gamma spectra. Each sample was exposed to 1800 s of radiation to provide reliable statistical results. Figure 2 illustrates the use of two collimators, each with a diameter of 0.3 cm, positioned in front of the source and detector. These collimators are made of lead and shaped like a disc with a thickness of 1.5 cm. The center of each collimator has a hole with a diameter of 0.3 cm. The distance between the point source and the sample was 10.3 cm, while the distance between the point source and the detector was 14.6 cm. Afterwards, the source was placed, and the initial gamma radiation, referred to as Io, was detected. Ultimately, the gamma counts for each material were determined at different thicknesses. To provide an unbiased evaluation, we calculated the relative counts (*I*/*I_o_*) for different thickness values of the samples and subsequently evaluated them.

## 3. Results and Discussion

### Characterization

**Figure 3:**
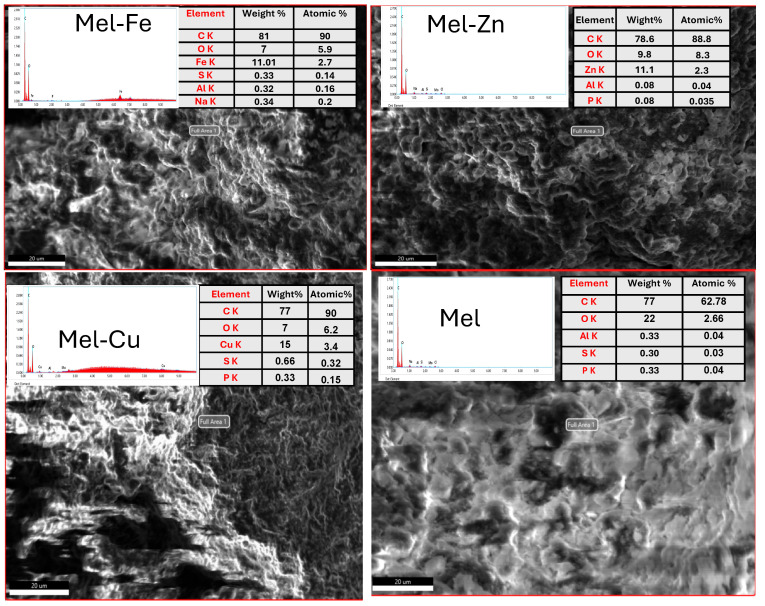

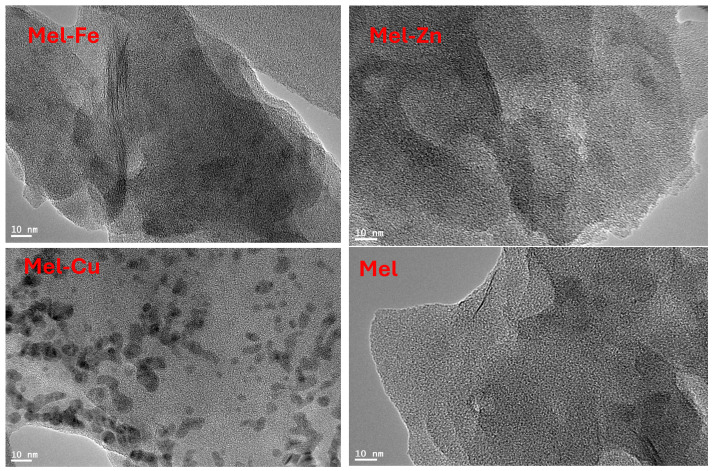
SEM micrographs and TEM images of eumelanin doped with Zn, Fe, and Cu ions.

SEM examination can provide insight into the structural organization of eumelanin. Figure 3 shows that all samples lacked a typical structural structure. Eumelanin samples were shown as small compasses and aggregated likes hetero-polymer consisting of diverse units. That decreased after being doped with transition metals (Fe, Zn, and Cu). The EDX analysis was applied to analyze the elemental and chemical composition of the eumelanin. The EDX results indicated the amplest elements with the superlative compositions for carbon atoms with Weights from 90% to 62.78%. The EDX results showed the highest concentrations of elements, particularly carbon atoms, which ranged from 90% to 62.78%. In contrast, the percentage of oxygen decreased significantly from 22% after doping with transition metals, dropping to 9.8% in the zinc sample (Mel-Zn). Meanwhile, the copper and iron samples each exhibited oxygen levels of approximately 7%. This indicates that metal–oxide bonds are likely forming due to the doping process with transition metals. TEM images analyzed the nano-size and cluster -shape of eumelanin. The structure appears to be sheet-like, with a rather uniform texture. Small granules are observed on the surface, indicating nanoparticle dispersal. Doping with metal ions results in a more defined, organized structure than pure eumelanin.

**Figure 4:**
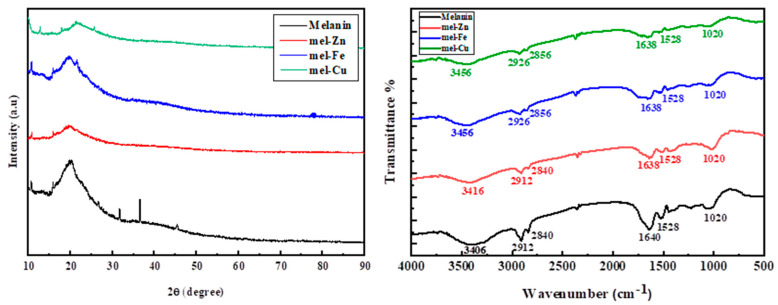
XRD patterns and FTIR spectra of eumelanin and Fe, Cu, and Zn ions.

As shown in Figure 4, The FTIR transmission spectra for pure eumelanin and metal-doped (Zn, Fe, and Cu) ions samples were analyzed in the range of 450-to-4000 cm^−^^1^. The spectrum of eumelanin reveals the presence of the catechol (O-H) group, characterized by a broad band at 3400 cm^−^^1^. Additionally, (C-H) stretching vibrations are indicated by bands at 2910 cm^−^^1^ and 2841 cm^−^^1^. The band observed at 1638 cm^−^^1^ is attributed to the presence of carboxylate (COO^−^) and/or aromatic (C=C) groups [24]. The signals in the range of 2800–3800 cm^−^^1^ arise from (O-H) and (N-H) stretching vibrations, reflecting the presence of both hydroxyl and amine groups. Furthermore, bands at 1042 cm^−^^1^ and 1530 cm^−^^1^ are associated with carboxylic acid and phenolic groups, while (C-H) vibrations contribute to the peak in the range of 671–698 cm^−^^1^. Notably, changes occur in the FTIR spectrum upon bonding of Zn, Fe, and Cu ions to natural eumelanin. The (O-H) group shifts from 3404 cm^−^^1^ to a higher wavenumber, indicating that the metal ions are interacting with the oxygen atoms in the functional groups of eumelanin. The peak intensity at 1640 cm^−^^1^, related to COO^−^, decreases and shifts slightly to 1638 cm^−^^1^, suggesting a bonding interaction between the metal ions and the ionized acid group. When Fe and Cu ions are present, the stretching modes associated with (C=C) or (COO^−^) at 2912 cm^−^^1^ and 2840 cm^−^^1^ shift to 2926 cm^−^^1^ and 2856 cm^−^^1^, respectively. In contrast, the FTIR spectra indicate that Zn ions do not significantly affect the structure of eumelanin. The XRD patterns show eumelanin before and after doping with Zn, Fe, and Cu ions. The XRD findings indicate that all of our samples have amorphous structures. The peak at 20° represents the production of eumelanin. Furthermore, there is a minor shift associated with a significant decrease in the strength of this peak in all co-doped samples, indicating that metal ions are connected to eumelanin. Furthermore, no additional Fe, Zn, or Cu peaks arise in the XRD patterns, indicating that the doped metal ions are well bound to the eumelanin molecule.

The densities of Fe, Cu, and Zn samples with eumelanin were calculated to determine the physical properties of the compounds and to measure radiation properties. Table 1 presents the experimental values of the calculated densities, revealing that the density of the samples varies depending on the component compositions and the type of material added to the eumelanin compound. In general, the addition of Fe, Cu, and Zn compounds to eumelanin clearly increased the density of the samples in different proportions; the Mel-Fe sample showed the highest density, with percentage increases of 6.01%, 4.98%, and 3.16% compared to the Mel density of the Mel-Fe, Mel-Cu, and Mel-Zn samples, respectively.

Energy-dispersive X-ray spectroscopy (EDX) is a key analytical technique for determining the elemental composition of samples, which is essential for various applications in radiation measurements. By identifying and measuring the elements present, EDX helps to theoretically calculate properties such as the mass attenuation coefficient. mass attenuation coefficient describes a material’s ability to attenuate X-rays or gamma rays, and allows comparison with experimental results. This analysis is critical for developing materials with specific radiation attenuation properties, such as those used in radiation shielding, nuclear science, and radiology. EDX is often used in conjunction with techniques such as scanning electron microscopy (SEM) to provide comprehensive material characterization and microstructural analysis, allowing researchers to optimize materials for use in radiation environments. Table 1 displays the mass distribution ratios of the constituent materials in the fabricated eumelanin samples. The mass distribution ratios of 15% were used for all Fe, Cu, and Zn samples that were added to the eumelanin. However, the ratios of 11% were used for the Fe and Zn samples after they were made for theoretical and experimental calculations.

The addition of iron (Fe), copper (Cu), and zinc oxide (Zn) to eumelanin leads to notable changes in both the linear and mass attenuation coefficients, suggesting that these additives enhance eumelanin’s ability to attenuate gamma radiation. The experimental results for four different gamma energies—59.5 keV (from ^214^Am), 661.7 keV (from ^137^Cs), 1173.2 keV, and 1332.5 keV (from ^60^Co)—were obtained by averaging the results for each sample in each photon energy.

**Figure 5:**
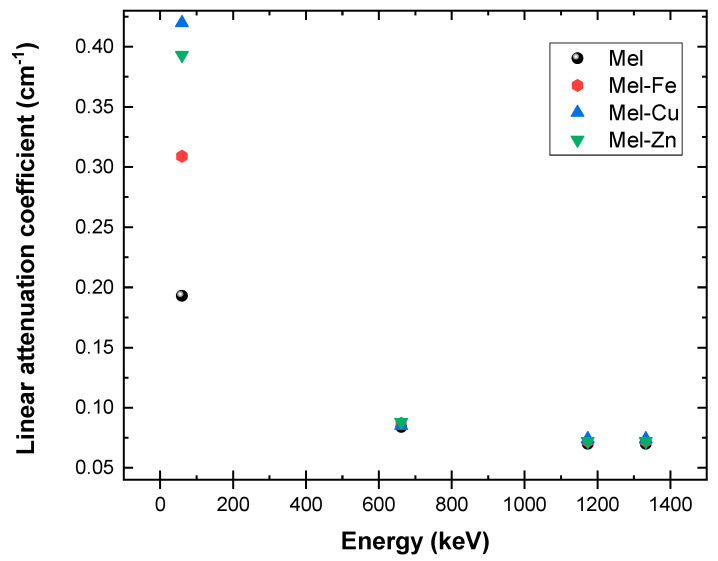
Measurement of the relationship between the linear attenuation coefficient and photon energy in fabricated eumelanin samples.

Figure 5 shows that the linear attenuation coefficient (*μ*) is higher for eumelanin samples with added Fe, Cu, and Zn compared to pure eumelanin (Mel), especially at lower gamma energies due to the higher atomic numbers of Fe, Cu, and Zn, which enhance photon absorption through the photoelectric effect. At 59.5 keV (^214^ Am), pure eumelanin (Mel) has a *μ* value of 0.193, while Mel-Fe increases significantly to 0.309, Mel-Cu shows an even higher value of 0.420, and Mel-Zn has an elevated *μ* value of 0.393. This substantial increase for Cu and Zn additives indicates their strong enhancement of photon absorption via the photoelectric effect, with Cu providing the most significant increase in attenuation at lower gamma energies. However, at higher gamma energies (661.7 keV from ^137^Cs, 1173.2 keV from ^60^Co, and 1332.5 keV from ^60^Co), the differences in *μ* between the additives are less pronounced. For instance, at 661.7 keV the *μ* values for Mel-Cu, Mel-Fe, and Mel-Zn are 0.085, 0.087, and 0.088, respectively, which are close to each other. At these higher energies, all samples, even pure eumelanin, have *μ* values that are pretty close to each other. This means that Compton scattering, not the photoelectric effect, is the main way that the samples interact, with the addition of Fe, Cu, and Zn having almost no effect on the attenuation.

An essential measure for evaluating the gamma-ray shielding efficiency of materials, regardless of their density, is the mass attenuation coefficient (*μ_m_*). As shown in Table 2, adding Fe, Cu, and Zn to eumelanin makes the mass attenuation coefficients much higher at lower gamma energies, like 59.5 keV, where the photoelectric effect is strongest. Eumelanin, in its pure form, has a *μ_m_* value of 0.153. However, the inclusion of Fe, Cu, and Zn raises the *μ_m_* values to 0.230, 0.316, and 0.302, respectively. This trend demonstrates that the addition of higher-atomic-number elements (Fe, Cu, and Zn) leads to an increase in photon absorption through the photoelectric effect. Among these additions, Cu and Zn exhibit the most notable increase. At gamma energies in the middle, like 661.7 keV, when Compton scattering is the main way that particles interact, the mass attenuation coefficients for all samples are very close to each other. They are Mel (0.067), Mel-Fe (0.065), Mel-Cu (0.064), and Mel-Zn (0.068). This suggests that the influence of the atomic number is less significant, and that electron density has a greater effect on attenuation in this range. At higher energy levels (1173.2 keV and 1332.5 keV from ^60^Co), the mass attenuation coefficients are even more similar across all samples, with values that are very close to each other. This observation indicates that the impact of the additives is insignificant when Compton scattering is the dominating factor. Furthermore, the error values seen in Table 2 are indicative of the experimental difficulties encountered when measuring attenuation coefficients at various energies and for different materials. Higher error rates were seen at lower gamma energies (59.5 keV), ranging from 0.014 to 0.028. This could be because the method is more sensitive to changes in sample preparation, thickness, and photoelectric interactions, especially when high-Z additions like Cu and Zn are used. By comparison, the reduced errors seen at intermediate and high energies (661.7 keV, 1173.2 keV, and 1332.5 keV) indicate more dependable results. These data are characterized by a decreased sensitivity of the primary interaction mechanisms (Compton scattering) to these parameters, with error values ranging from 0.003 to 0.005. 

**Figure 6:**
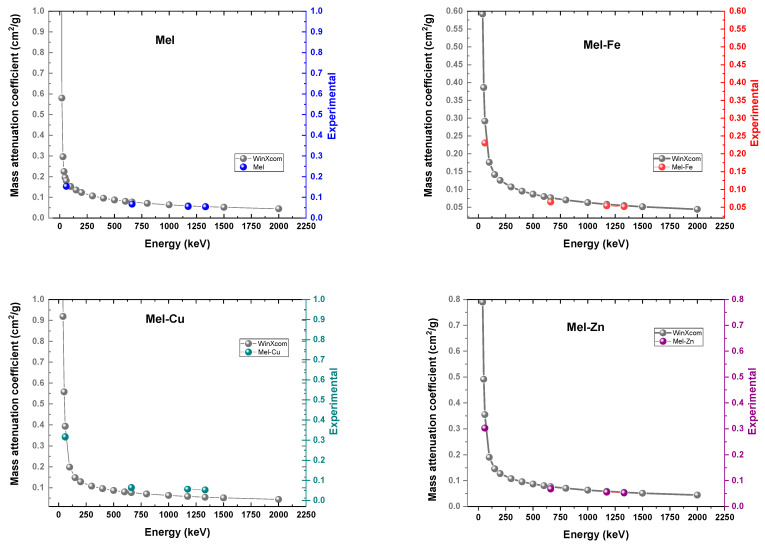
Theoretical (WinXCOM) and experimental mass attenuation coefficients for eumelanin samples.

Figure 6 displays the quantitative mass attenuation coefficients obtained from experimental measurements in our investigation within the energy range of 59.5 keV–1332.5 keV, together with the theoretical values within the broader range of 10 keV–2000 keV. The observed trend is that the *μ_m_* exhibits a rapid drop as the photon energy increases, while it increases with the Weight of the additive material (Fe, Cu, and Zn). It is clear that the *μ_m_* is affected by both the filler mass and photon energy. This aligns with the findings reported in the study conducted by [34]. These findings indicate that the inclusion of Fe, Cu, and Zn is very efficient in reducing the intensity of low-energy gamma rays. This makes these composites appropriate for applications such as medical imaging or radiation shielding, where low-energy photons are common. Nevertheless, when dealing with higher-energy gamma rays, where Compton scattering is the primary factor, the impact of these additions on mass attenuation is insignificant. In such cases, other material characteristics, such as mechanical strength or cost, may become more crucial in the choice of materials for radiation shielding. This study is very important for making eumelanin-based composites that are specifically designed for certain radiation shielding tasks based on the gamma rays that are used.

The differences in the *μ_m_* of the fabricated eumelanin samples, as presented in Table 3, can be attributed to many aspects associated with the samples’ characteristics, the settings in which the experiments were conducted, and the constraints of the theoretical model. For example, the sample Mel exhibits a percentage deviation of 15.00% at 59.5 keV, which then drops to 1.82% at 1332.5 keV. The fact that the deviation decreases as the energy level rises suggests that lower photon energies are more affected by factors like sample composition, uneven density, and possible contaminants that can change attenuation measurements. Furthermore, Mel-Fe demonstrates a significant divergence of 21.23% at 59.5 keV and 5.45% at 1332.5 keV, suggesting a considerable influence of Fe inclusion on photon interactions, especially at lower energy levels when photoelectric absorption is the dominant factor. The different deviations seen in different samples, like Mel-Cu (19.59% at 59.5 keV and 3.70% at 1332.5 keV) and Mel-Zn (14.69% at 59.5 keV and 3.64% at 1332.5 keV), show how the different atomic numbers and cross-sections of the elements used have an effect. The greater disparities observed at lower energy levels may be attributed to the heightened probability of scattering and absorption phenomena, as well as the partial penetration of photons. Conversely, at higher energy levels a tighter correspondence between experimental and theoretical values suggests a diminished influence of these secondary effects. Furthermore, the observed values are crucially influenced by experimental parameters such as detector efficiency, source-to-sample distance, and beam alignment. These factors contribute to minor variations of WinXCOM’s theoretical predictions, which assume optimal situations.

Table 4 shows that comparing *µ_m_* at 59.5 keV is crucial, as this low gamma energy is where the photoelectric effect dominates, making it highly relevant for evaluating the efficiency of shielding materials. With a *µ_m_* of 0.153 cm^2^/g (experimental) and 0.180 cm^2^/g (theoretical), eumelanin showed a lot of promise as a radiation shield in this study. Compared to widely used polymeric shielding materials such as poly(methyl methacrylate) (*µ_m_* = 0.109 cm^2^/g) and polypropylene (0.126 cm^2^/g), eumelanin achieves approximately 40%- and 21%-higher attenuation, respectively, highlighting its superior performance for use in radiation panels and medical dosimetry tools, where conventional materials often fall short in low-energy gamma shielding. Also, eumelanin works well against aromatic polymers that are known for having high attenuation properties. For example, phenol-formaldehyde resin = 0.173 cm^2^/g and polycarbonate = 0.172 cm^2^/g are only 11%- and 12%-different from eumelanin. These materials are commonly used in radiation detector housings and industrial shielding, but they lack the biocompatibility and eco-friendly properties that eumelanin offers. Unlike these conventional materials, eumelanin provides dual functionality, combining effective gamma-ray attenuation with free radical scavenging properties, making it highly valuable for biomedical applications, including wearable protective garments, medical implants, and device coatings, where both radiation protection and biological safety are critical.

**Figure 7:**
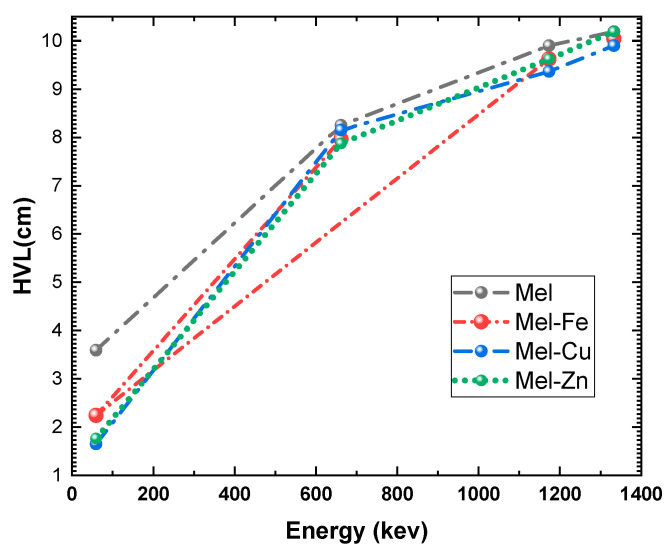
Half-value layer (HVL) of the fabricated eumelanin samples at photon energies of 59.5 keV, 661.7 keV, 1173.2 keV, and 1332.5 keV.

The HVL values of eumelanin polymer samples with added Fe, Cu, and Zn were obtained at 59.5 keV, 661.7 keV, 1173.2 keV, and 1332.5 keV. At 59.5 keV, the half-value layer (HVL) measurements for Mel, Mel-Fe, Mel-Cu, and Mel-Zn were 3.591 cm, 2.243 cm, 1.650 cm, and 1.763 cm, respectively, as shown in Figure 7. Copper exhibited the greatest attenuation at 54.0%, followed by zinc at 50.9% and iron at 37.6%, signifying an increased photoelectric absorption attributable to their elevated atomic numbers. At 661.7 keV, the half-value layer (HVL) measurements were 8.250 cm for Mel, 7.966 cm for Mel-Fe, 8.153 cm for Mel-Cu, and 7.875 cm for Mel-Zn. Zinc had the most attenuation (4.5%) because it has the best atomic number and density for Compton scattering. At 1173.2 keV, the half-value layer (HVL) measurements were 9.900 cm (Mel), 9.625 cm (Mel-Fe), 9.365 cm (Mel-Cu), and 9.625 cm (Mel-Zn), with copper exhibiting the most significant drop at 5.4%. At 1332.5 keV, the half-value layer (HVL) measurements were 10.191 cm (Mel), 10.043 cm (Mel-Fe), 9.900 cm (Mel-Cu), and 10.191 cm (Mel-Zn), with copper exhibiting the lowest HVL value at 2.9%. Copper doping consistently showed better effectiveness across all energy levels, establishing it as the most effective dopant, but zinc and iron also enhanced shielding.

**Figure 8:**
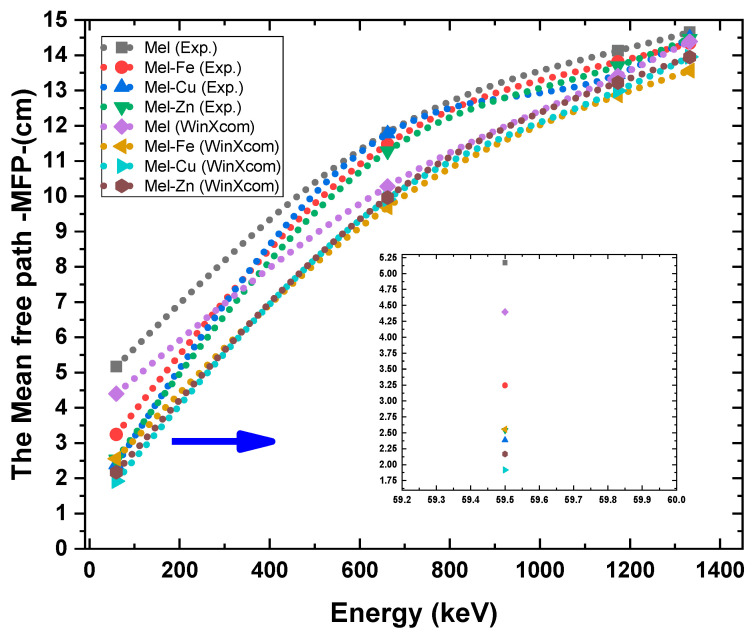
The mean free path (MFP) for the Mel, Mel-Fe, Mel-Cu, and Mel-Zn samples at photon energies of 59.5 keV, 661.7 keV, 1173.2 keV, and 1332.5 keV.

The mean free path (MFP) is an essential quantity in radiation shielding that quantifies the average distance covered by a photon within a material prior to its interaction. The mean free energy (MFP) values for eumelanin (Mel) and its composites with iron (Fe), copper (Cu), and zinc (Zn) at different photon energies (59.5 keV, 661.7 keV, 1173.2 keV, and 1302.5 keV) are shown in Figure 8. The results show that adding Fe, Cu, and Zn to eumelanin greatly lowers the MFP at lower energy levels, which suggests better attenuation properties. At 59.5 keV, pure eumelanin has an MFP of 5.172 cm. This value significantly reduces to 3.244 cm for Mel-Fe, 2.385 cm for Mel-Cu, and 2.540 cm for Mel-Zn. The observed decrease shows that some additions, especially copper, make it much easier for photons to be absorbed by the photoelectric effect. This results in fewer mean free pathways and better shielding at low energy levels. The experimental findings are in excellent agreement with the theoretical predictions (WinXCOM), with minor deviations likely caused by experimental circumstances and sample preparation.

The variations in the MFP among the samples become less significant at higher photon energies (661.7 keV, 1173.2 keV, and 1332.5 keV) when Compton scattering is the dominant factor. At an energy level of 661.7 keV, the MFP values exhibit considerable proximity—11.810 cm for Mel, 11.478 cm for Mel-Fe, 11.775 cm for Mel-Cu, and 11.280 cm for Mel-Zn—demonstrating little deviation. This behavior persists at 1173.2 keV and 1332.5 keV, when the MFP values for all samples approach a range of 13–15 cm. This suggests that the additives have a reduced influence on the attenuation characteristics at these higher energy levels. The findings indicate that the addition of Fe, Cu, and Zn to eumelanin greatly enhances its ability to protect against lower energy levels. Among these, Cu exhibits the highest level of efficacy, but its impact decreases at higher photon energies. 

**Figure 9:**
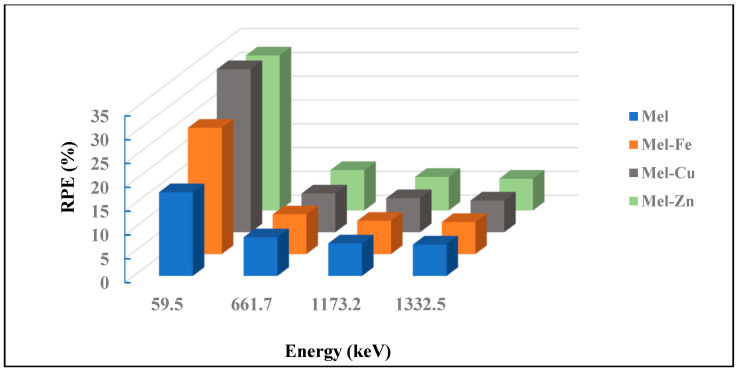
Relationship between RPE and energy in the Mel, Mel-Fe, Mel-Cu, and Mel-Zn samples.

The radiation protection efficiency (RPE) values for eumelanin (Mel) and its composites with Fe, Cu, and Zn indicate significant variations in shielding effectiveness across different photon energies, as shown in Figure 9. At a low energy of 59.5 keV, pure eumelanin exhibits an RPE of 17.581%, which is notably increased by adding Fe, Cu, and Zn, resulting in RPE values of 26.529%, 34.251%, and 32.545%, respectively. This shows that Cu offers the highest improvement in radiation protection at this energy level. As the photon energy increases to 661.7 keV, the RPE values decrease, showing less differentiation between the samples—8.119% for Mel, 8.344% for Mel-Fe, 8.142% for Mel-Cu, and 8.483% for Mel-Zn—indicating a modest enhancement with these additives. At the even higher energies of 1173.2 keV and 1332.5 keV, the RPE values continue to converge, with Mel having 6.833% and 6.597%, Mel-Fe having 6.982% and 6.733%, Mel-Cu having 7.162% and 6.668%, and Mel-Zn having 7.041% and 6.676%, respectively. These results suggest that while Fe, Cu, and Zn significantly improve the radiation protection efficiency of eumelanin at lower energies, their impact becomes more uniform and less pronounced at higher energies.

**Figure 10:**
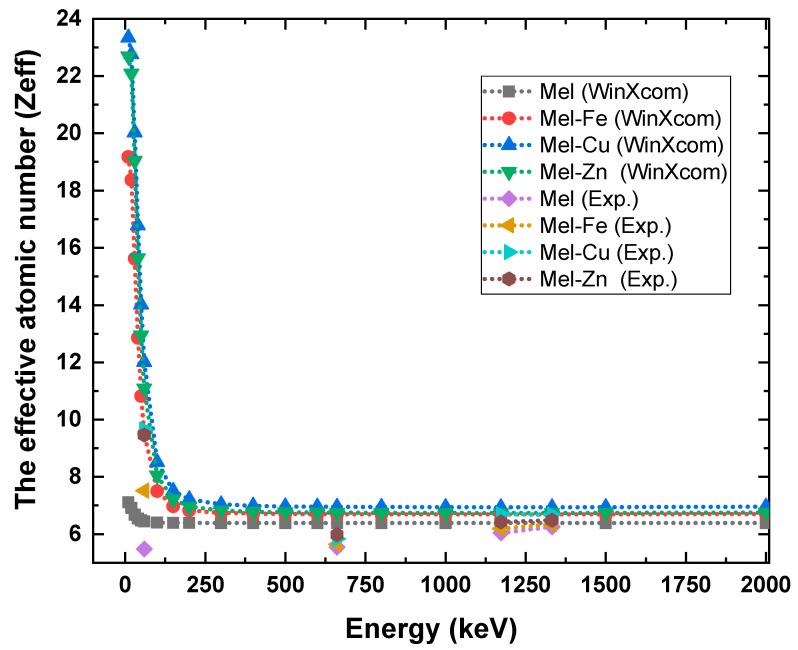
The effective atomic number (*Z_eff_*) of the fabricated eumelanin samples.

As shown in Figure 10, the effective atomic number (*Z_eff_*) results for pure eumelanin (Mel) and its composites with Fe, Cu, and Zn show a significant increase in radiation shielding effectiveness due to metal doping, particularly at lower photon energies. Figure 10 shows that at 59.9 keV the *Z_eff_* increases from 5.478 for pure eumelanin to 7.514 for Mel-Fe, 9.655 for Mel-Cu, and 9.462 for Mel-Zn, demonstrating that Cu provides the highest enhancement. As the photon energy increases, the *Z_eff_* values for all samples converge, indicating less variation between the doped and undoped samples. At 661.6 keV, the *Z_eff_* values are 5.544 for Mel, 5.645 for Mel-Fe, 5.831 for Mel-Cu, and 5.980 for Mel-Zn, while at 1332 keV they are 6.253, 6.342, 6.681, and 6.483, respectively. This convergence suggests that the impact of metal additives on *Z_eff_* is more pronounced at lower energies, where radiation shielding improvements are more significant, highlighting the potential of Cu-doped eumelanin for optimized shielding applications.

## 4. Conclusions

This study demonstrates that doping eumelanin with metals like iron (Fe), copper (Cu), and zinc (Zn) significantly improves its gamma radiation shielding properties, particularly at lower photon energies. The results show that both the linear (*μ*) and mass attenuation coefficients (*μ_m_*) are notably higher for doped samples compared to pure eumelanin. For instance, at a low energy of 59.5 keV pure eumelanin exhibits a *μ* value of 0.193, which increases to 0.309 for Mel-Fe, 0.420 for Mel-Cu, and 0.393 for Mel-Zn. Similarly, the mass attenuation coefficient increases from 0.153 cm2/g for pure eumelanin to 0.230, 0.316, and 0.302 cm^2^/g for Mel-Fe, Mel-Cu, and Mel-Zn, respectively. These increases are attributed to the higher atomic numbers of Fe, Cu, and Zn, which enhance photon absorption via the photoelectric effect. However, at intermediate and higher energies (661.7 keV-to-1332.5 keV), where Compton scattering dominates, the differences in attenuation coefficients among the samples diminish, indicating a reduced impact of the metal additives.

Furthermore, the mean free path (MFP) and radiation protection efficiency (RPE) results further demonstrate the benefits of metal doping at a low photon energy. At 59.5 keV, the MFP is reduced from 5.172 cm for pure eumelanin to 3.244 cm for Mel-Fe, 2.385 cm for Mel-Cu, and 2.540 cm for Mel-Zn, indicating improved photon absorption and enhanced shielding effectiveness. The RPE values at the same energy level show a substantial increase, with pure eumelanin having an RPE of 17.581%, which rises to 26.529% for Mel-Fe, 34.251% for Mel-Cu, and 32.545% for Mel-Zn. According to these results, all three additives make eumelanin a better shielding material at low-energy gamma rays, but copper (Cu) does the best overall, showing the highest improvement in both attenuation coefficients and radiation protection efficiency.

## Figures and Tables

**Table 1:** Composition and calculated density of fabricated eumelanin samples.

Samples	Element Composition (%)									Density (g/cm^3^)
	C	O	Al	S	P	Fe	Cu	Na	Zn	
Mel	77.00	22.00	0.33	0.30	0.33	-	-	-	-	1.264
Mel-Fe	81.00	7.00	0.32	0.33	-	11.01		0.34	-	1.340
Mel-Cu	77.00	7.00	-	0.66	0.33	-	15.00	-	-	1.327
Mel-Zn	78.60	9.80	0.08	0.08	0.08	-	-	-	11.10	1.304

**Table 2:** Linear and mass attenuation coefficients of fabricated eumelanin samples measured experimentally.

Samples	^214^Am (59.5 keV)			^137^Cs (661.7 keV)			^60^Co (1173.2)			^60^Co (1332.5 keV)		
	*μ*	*μ_m_*	Error (±%)	*μ*	*μ_m_*	Error (±%)	*μ*	*μ_m_*	Error (±%)	*μ*	*μ_m_*	Error (±%)
Mel	0.193	0.153	0.014	0.084	0.067	0.008	0.070	0.056	0.005	0.068	0.054	0.004
Mel-Fe	0.309	0.230	0.017	0.087	0.065	0.007	0.072	0.054	0.006	0.069	0.052	0.004
Mel-Cu	0.420	0.316	0.028	0.085	0.064	0.010	0.074	0.056	0.004	0.070	0.052	0.003
Mel-Zn	0.393	0.302	0.024	0.088	0.068	0.007	0.072	0.056	0.006	0.068	0.053	0.005

**Table 3:** Comparison of experimental and WinXCOM results based on the mass attenuation coefficients of the fabricated eumelanin samples.

Samples	Energy (keV)	Mass Attenuation Coefficients (cm^2^/g)		
		Experimental	Theoretical (WinXCOM)	Percentage Deviation%
Mel	59.5	0.153	0.180	15.00
	661.7	0.067	0.077	12.99
	1173.2	0.056	0.059	5.08
	1332.5	0.054	0.055	1.82
Mel-Fe	59.5	0.230	0.292	21.23
	661.7	0.065	0.077	15.58
	1173.2	0.054	0.058	6.90
	1332.5	0.052	0.055	5.45
Mel-Cu	59.5	0.316	0.393	19.59
	661.7	0.064	0.076	15.79
	1173.2	0.056	0.058	3.45
	1332.5	0.052	0.054	3.70
Mel-Zn	59.5	0.302	0.354	14.69
	661.7	0.068	0.077	11.69
	1173.2	0.056	0.058	3.45
	1332.5	0.053	0.055	3.64

**Table 4:** Mass attenuation coefficient values compared to other materials studied at 59.5 keV in the literature.

Study	Study Theme	Material	$\mu_{m}$ $\left(cm^{2}/g\right)$
Singh et al. [35]	Simulation (MCNP)	phenol-formaldehyde resin (*ρ* =1.36 g/cm^3^)	0.173
	Simulation (MCNP)	Polycarbonate (*ρ* = 1.22 g/cm^3^)	0.172
Kucuk et al. [36]	Experimental	Poly(methyl methacrylate) (*ρ* = 1.18 g/cm^3^)	0.109
	Experimental	Polypropylene (*ρ* = 0.946 g/cm^3^)	0.126
	Experimental	Polyethylene (*ρ* = 0.920 g/cm^3^)	0.112
This study	Experimental	eumelanin (Mel)	0.153
	Theoretical (XCOM)	eumelanin (Mel)	0.180
	Experimental	Mel-Fe	0.230
	Experimental	Mel-Cu	0.316
	Experimental	Mel-Zn	0.302

## Data Availability

Data are contained within the article.
